# Editorial: Quality control for efficacy and safety of herbal medicinal products

**DOI:** 10.3389/fphar.2023.1162698

**Published:** 2023-05-25

**Authors:** Wing Lam, Wei Cai, Yong Li, Zhichao Xu, JianBo Yang

**Affiliations:** ^1^ Department of Pharmacology, Yale University, New Haven, CT, United States; ^2^ School of Pharmaceutical Sciences, Hunan University of Medicine, Huaihua, China; ^3^ Institute of Materia Medica, Chinese Academy of Medical Sciences and Peking Union Medical College, Beijing, China; ^4^ College of Life Science, Northeast Forestry University, Harbin, China; ^5^ National Institutes for Food and Drug Control, Beijing, China

**Keywords:** quality control, efficacy, safety, herbal medicinal products, DNA barcode, UPLC-HRMS, data mining

## Introduction

Herbal medicines have a long-standing history in human culture, serving as the foundation for various traditional medical systems, including Traditional Chinese Medicine, Ayurveda, Unani, and Siddha. In Asia and some other countries, plant-based medicines are often regarded as mainstream treatments for a range of complex diseases and symptoms. However, in many developed Western nations, such as the United States, herbal products are predominantly marketed as dietary supplements, which are not permitted to make claims about diagnosing, curing, mitigating, treating, or preventing diseases.

Although botanical medicines are often perceived as safer due to their natural, plant-derived origins, there have been numerous reports of toxicity among cancer patients using herbal medicines (Olaku and White, 2011). To gain acceptance as legitimate medicine within the Western medical framework, researchers and developers must address quality control Research Topic and enhance our understanding of the safety profile of herbal medicines through rigorous scientific approaches. This will help ensure that these products meet the guidance and regulatory standards of different countries. Adhering to the Botanical Drug Development Guidance for Industry, the United States FDA approved a select few herbal products for sale as prescription drugs. Notable examples include sinecatechins (Veregen^®^) and crofelemer (Mytesi™).

Over the past 20 years, significant progress has been made in developing and adapting numerous cutting-edge techniques to address the efficacy and safety of herbal medicinal products. In this Research Topic Research Topic, we have included nine original research articles that cover various aspects of quality control for the efficacy and safety of herbal medicinal products. We hope that these articles will enhance our understanding of the importance of quality control in the realm of botanical medicinal products.

To develop herbal medicine, the correct herb species must be identified as the first step. Therefore, developing methods for accurate herbal authentication is foundational. In the past, experienced herbalists were relied on for selection based on morphology, color, smell, and taste. In addition to these traditional methods, which can be too subjective, technological advances that provide much more precise data have been invented and applied to herbal authentication. Mahima et al. provide very detailed information about using DNA barcoding techniques for herbal authentication. The Mahima et al. article introduces the history and the trend of DNA barcoding for the herbal industry (Mahima et al.). Mahima et al. collected many examples in which DNA barcoding techniques were applied to the authentication and detection of contaminants. In addition, Mahima et al. cover the view of different countries’ Pharmacopoeia or regulatory agencies on DNA barcoding for herbal products. Furthermore, Mahima et al. also discuss the most updated DNA barcoding methodologies (Mahima et al.). Universal DNA barcoding based on nuclear DNA has limitations in differentiating species of some plant families, for example, *Bidens* species. Wu et al. analyze DNA sequences from 12 chloroplast and eight mitochondrial genomes of five species and one variety of Bidens using bioinformatics (Wu et al.). They reveal that complete chloroplast genomes could be used as a super barcode to authenticate Bidens species accurately (Wu et al.). In addition, highly variable regions of trnS-GGA-rps4 could potentially be used as a specific barcode to identify Bidens species (Wu et al.).

Besides plant species authentication, herbal products must also meet contamination standards set by their respective country government agencies. Pesticide residues with known harmful linkages to health found in herbal products are a serious safety concern. Some farmers are even using new pesticides to avoid detection. Wang et al. describe using high-performance liquid (HPLC-MS/MS) and gas (GC-MS/MS) chromatography to effectively detect 168 pesticides in 1,017 samples of 10 herbs that are commonly sold in markets (Wang et al.). They report that the pesticide levels in Chinese Herbal Medicines of the same type had varied distribution. In addition, Wang et al. use bioinformatics to assess short-term and long-term intake risk and cumulative dietary risk (Wang et al.).

Differences in seasonal and environmental factors have demonstrated that separate batches of the same herb may have different efficacy. Furthermore, herbal medicines contain multiple active chemicals, and their metabolism by the human body could be very different depending on their interactions Innovative methods that could effectively identify and quantify multiple active metabolites and/or contaminants, such as pesticides, should be very useful in assessing efficacy and toxicity. One such tool, UHPLC-HRMS (ultra-high-performance liquid chromatography-high resolution mass spectrometry), is a very powerful instrument that has very high sensitivity and precision for molecular identification and quantification. By introducing the advantages of UPLC-HRMS and its specific applications, such as chemical characterization, determination of TCM components, chemical fingerprint analysis, identification of the authenticity of TCMs, and identification of illegal additives, Ma et al. discuss how to apply this technology to study metabolites. They also collected some examples in which researchers used UHPLC-HRMS technology with PCA and OPLS-DA statistical methods to study mechanisms of action of various Traditional Chinese Medicines (TCM) (Ma et al.).

For the past two decades, steady progress has been made in generating and accumulating data on herbal medicine. This information is collected in publicly available online databases. Applying computational methods to take advantage of this big data could facilitate the quality control of herbal medicines. Using network pharmacology and molecular docking methods, An et al. identify toxic components of Fuzi and its potential targets that are associated with neurotoxicity. They also validate that Aconitine in Fuzi could affect mitochondrial function, induce apoptosis, and inhibit MARK and AKT phosphorylation in neuron cells. An et al. also confirm that Aconitine could reduce the number of normal hippocampal neurons by inducing apoptosis. In another study, Chen et al. use data processing techniques to identify active compounds from herbal medicine (Chen et al.). In their report, they rank 20 potential Quality-markers (Q-marker) of Jiuzhi Dahuang Wan (JZDHW: processed Rhubarb) using non-targeted/targeted data mining technologies and the time-concentration curve (AUC) pooled methods (Chen et al.). Ultimately, the compound rhein was selected as a Q-marker, and it was validated in mice that it could reduce LPS-induced pneumonia by inhibiting IL1β and IL6 production and pathological changes in lung tissue (Chen et al.). Chen et al. also find that rhein could be used to predict the overall anthraquinone metabolism of JZDHW *in vivo*.

In this Research Topic, we also selected three articles focusing on Polygonum multiflorum Thunb (PM) (the dried root of PM, or Polygoni Multiflori Radix) because it has been reported in the past few decades to occasionally cause hepatoxicity. PM has a long usage history in treating different diseases, but its preparation can have very different properties; raw PM is better suited for detoxifying and anti-swelling, while processed PM has immunomodulating, tonifying properties. According to the TCM application, processed (stewed with black bean juice, steamed with black bean juice, and steamed with water) PM preparations are less toxic to humans. To investigate this, Gu et al. compare polysaccharide changes in raw PM and processed PM. They find that processed PM could reduce polysaccharides Mw, indicating that polysaccharides degradation occurred somewhere during the processing (Gu et al.). They also find that processing increases the molar ratio of Glc/GalA in processed PM polysaccharides. Therefore, the Mw and Glc/Gal A ratios could serve as quality control markers for processed PM. They reveal that both processed PM and raw PM could promote the cell viability of RAW264.7 macrophages, but processed PM polysaccharides exhibited stronger induction on IL6, TNFa, and iNOS mRNA expression than raw PM (Gu et al.). Their result indicates that the changes in polysaccharides during the processing could affect its immunomodulatory activity.

Articles by Song et al. and Kang et al. both aim to address PM’s reported hepatotoxicity. Song et al. integrate metabolomics and the pseudo-targeted spectrum–effect relationship to capture potential hepatotoxic components in PM. Song et al. compare compounds between raw PM and processed PM based on tentative UPLC-Q-TOF-MS identification and toxicity and attenuation methods. Using three mathematical models (gray relational analysis, orthogonal partial least squares analysis, and back propagation artificial neural network methods), they correlate the quantity of proposed pseudo-targeted MS of 16 differential components from 50 PM batches and the hepatocytotoxicity of 50 batches of PM. Their analysis pinpoints three distinct components (emodin dianthrones, emodin-8-O-β-D-glucopyranoside, PM 14–17) that could serve as PM toxicity markers (Song et al.).

To identify toxic components in PM, Kang et al. study the hepatotoxicity impact of PM’s anthraquinones on bile acid (BA) homeostasis in mice. By comparing the impact of PM extract to individual anthraquinones on liver function, they reveal that physcion and PM extract could alter BA metabolism and the expression of Bsep and Mrp2 during treatment (Kang et al.). By examining the metabolism of bile acids and protein/mRNA expression of bile salt export pump (Bsep) and multidrug resistance-associated protein 2 (Mrp2), Kang et al. provide a new mechanism of action to elucidate how liver function can be affected by PM.

Due to space constraints in this Research Topic Research Topic, we are only able to feature nine exceptional articles that focus on herbal authentication, metabolite/chemical identification, big data mining, and analysis. These articles present innovative approaches for improving the quality control, efficacy, and safety of herbal medicinal products. Many other Research Topic related to the efficacy and safety of herbal medicinal products, such as interactions between standard treatments and herbal/natural products, will be included in “*Quality control for efficacy and safety of herbal medicinal products: volume II.*” We look forward to presenting more compelling articles in our upcoming Research Topic Research Topic.
